# Effects of far-infrared radiation on the nervous system and their potential

**DOI:** 10.20407/fmj.2026-011

**Published:** 2026-09-25

**Authors:** Naoki Aizu, Takumi Kito, Kazuhiro Nishii, Kouji Yamada

**Affiliations:** 1 Major in Health Sciences, Graduate School of Health Sciences, Fujita Health University, Toyoake, Aichi, Japan; 2 Faculty of Rehabilitation, School of Health Sciences, Fujita Health University, Toyoake, Aichi, Japan; 3 Department of Physical Therapy, Faculty of Health Sciences, Kinjo University, Hakusan, Ishikawa, Japan

**Keywords:** Far-infrared radiation (FIR), Neurological disease, Dysmnesia, Nerve regeneration, Neuronal death

## Abstract

Far-infrared radiation (FIR; 3–1000 μm) is characterized by its strong tissue penetration and efficient heat conduction properties. Specifically, wavelengths within the range of 8–14 μm—often referred to as growth rays—are efficiently absorbed by living organisms, generating pronounced thermal effects. These wavelengths closely correspond to the natural vibrational wavelength of water molecules, thus enabling effective resonance and energy absorption. This property has led to their widespread use as a therapeutic tool in biomedical and healthcare applications. Because living organisms contain a substantial proportion of water, FIR can penetrate deeply into tissue, influencing various molecules through resonance with water molecules and modulating molecular mechanisms, thereby eliciting a range of physiological effects. The effects of FIR have been examined across diverse disciplines. The present review focuses on the effects of FIR on the nervous system. It compiles and discusses findings from previous studies that have used animal models of nervous system diseases to explore the direct effects of FIR on various aspects of the nervous system, including memory impairment, cognitive function, neural regeneration, sleep, anti-inflammatory effects, and stress relief.

## Introduction

Infrared radiation (IR) is a type of electromagnetic wave, similar to invisible light and radio waves, characterized by wavelengths that exceed those of visible light. The International Commission on Illumination categorizes IR into three bands based on wavelength: near-IR (NIR: 0.78–1.4 μm), mid-IR (MIR: 1.4–3 μm), and far-IR (FIR: 3–1000 μm).^1^ FIR is highly thermally conductive and can penetrate various materials, including water, plastics, paints, and food, which typically absorb infrared radiation within the range of 2–20 μm. In particular, wavelengths known as growth rays (8–14 μm) are efficiently absorbed by living organisms, resulting in thermal effects.^2^ Consequently, FIR has gained widespread acceptance as a beneficial therapeutic tool in biomedical and healthcare applications.^3,4^ There are three ways in which heat is transferred: conduction, convection, and radiation; FIR is transmitted only through radiation. Heat warms the surface of a substance, whereas FIR reaches deep cells and tissues through radiation, thus warming the inside of the substance. The deep penetration power, acting on the deep parts of the body, is proportional to the wavelength. FIR penetrates deep into the body, causing resonance with water molecules in the body; the vibrational energy from this resonance generates thermal energy. Because the wave frequency of FIR is almost the same as that of the molecules that make up the human body, a strong resonance effect occurs.^2,5^

Growth rays are efficiently absorbed by living organisms, generating pronounced thermal effects. FIR therapy uses longer wavelengths than NIR therapy, and FIR is absorbed and emitted by the body as blackbody radiation (FIR absorber, 3–50 μm, peak output at 9.4 μm).^2,5^ Moreover, FIR therapy is presumed to have no side effects, and has garnered substantial attention for its health benefits.^6,7^ Various methods have been used in research into FIR, including FIR-emitting panels such as ceramic plates, special lamps that irradiate the area from above, and fabrics with filaments that are impregnated with FIR-emitting nanoparticles.^2^ The intensity, duration, and frequency of FIR are not standardized.

Research into the effects of FIR on living organisms has been extensively documented across various fields, with numerous reviews published in this area. Notably, many studies have directly examined the effects of FIR on the nervous system, often using animal disease models for conditions such as Alzheimer’s disease (AD), Parkinson’s disease (PD), multiple sclerosis (MS), depression, and spinocerebellar ataxia (SCA) etc. (Table 1). The findings of these studies have highlighted the impact of FIR on various aspects of the nervous system, including memory impairment, cognitive function, neural regeneration, sleep quality, inflammation reduction, and stress relief.

In the present review, PubMed was searched using the keywords “Far infrared rays and brain,” “Far infrared rays and neuron,” “Far infrared rays and metabolism,” and “Far infrared rays and stress.” The results were reviewed collaboratively by the co-authors, and animal and cell culture experiments were considered. Subsequently, another PubMed search was performed using the additional keywords “Far infrared and Alzheimer’s,” “Far infrared and promotes,” and “Far infrared rays glucose.” The results were reviewed collaboratively by the co-authors, and articles consistent with the main theme and objectives of the review article were selected. The goal is to compile research reports demonstrating that far-infrared radiation directly affects the nervous system and exhibits beneficial effects. Although the present review was focused on newer literature (from 2000 onward), older literature was identified from previous review articles, and those deemed necessary were included as references. The period around 2000 was a turning point where research methods and data collection techniques changed dramatically in all fields, due to the spread of the internet, the digitization of databases, and the full-scale implementation of genome analysis. While it is usually desirable to summarize new findings from the last 5 to 10 years, this study targeted the period from when reports on the effects of far-infrared radiation on genetically modified animal models began to increase until the present day. The purpose of this review was to provide a comprehensive summary of research studies that have explored the direct effects of FIR on the nervous system.

## Direct effects of FIR on the nervous system

### Effects of FIR on memory

Several studies using animal models have demonstrated the efficacy of FIR in improving memory impairment. In a study involving protein kinase C delta (PKCδ) knockout mice, exposure to FIR mitigated methamphetamine-induced impairments in recognition memory. This effect was achieved through upregulation of the M1 muscarinic acetylcholine receptor, the induction of glutathione dependent on NF-E2-related factor 2 (Nrf2), and the phosphorylation of extracellular signal-regulated kinase (ERK)1/2. These outcomes were attributed to the inhibition of PKCδ phosphorylation by bryostatin 1.^8^ In an experimental model examining methamphetamine-induced impairments in recognition memory in male mice repeatedly treated with methamphetamine, FIR prevented methamphetamine-induced memory deficits. This protective effect occurred through activation of the Nrf2-dependent glutathione synthesis pathway and ERK1/2 signaling, resulting from inhibited expression of the gene encoding PKCδ.^9^

A study using TgCRND8 transgenic mice, which serve as a model for AD, demonstrated that FIR effectively reduces amyloid β (Aβ) burden and tau hyperphosphorylation. It also suppresses neuroinflammation and improves cognitive dysfunction in TgCRND8 mice. This process appears to involve the restoration of oxidation-related proteins through modulation of the Janus kinase 2 (JAK2)/signal transducer and activator of transcription 3 (STAT3) pathway and the Nrf2/heme oxygenase-1 pathway.^10^ Following FIR, 5xFAD AD model mice exhibit increased expression levels of nerve growth factor (NGF) in the hippocampus and mature brain-derived neurotrophic factor in the cerebral cortex. The depletion of neurotrophic factors can impair various neuronal functions, including the degeneration of synapses, dendrites, and axons.^11^ FIR also reduces Aβ accumulation (a key feature of AD neuropathology), mitigates neuroinflammation, restores expression of the presynaptic protein synaptophysin, and enhances learning and memory deficits in AD model mice.

FIR also activates the mitochondrial oxidative phosphorylation pathway, leading to increased ATP production. Elevated intracellular ATP levels then promote the release of extracellular ATP from Aβ-stimulated microglia, thereby enhancing Aβ phagocytosis through the phosphoinositide 3-kinase (PI3K)/mechanistic target of rapamycin (mTOR) pathway, which facilitates Aβ clearance.^12^ In a study using *APP/PS1* mice—another type of AD model knock-in mice—individuals that received MIR with a peak wavelength of 7.7–10 μm demonstrated improved learning and memory abilities alongside a reduced Aβ plaque burden in the brain. Moreover, the composition of the gut microbiota returned to normal (i.e., to that of wild-type mice). However, this was the result of MIR rather than FIR. Collectively, these findings suggest that photobiomodulation is a novel, non-invasive, and promising approach to AD treatment. Additionally, they suggest that the gut microbiota may potentially be used as a target for AD via the brain–gut–skin axis.^13^

### Effects of FIR on nerve regeneration

In a study into nerve regeneration, FIR in NGF-treated PC12 cells (which exhibit characteristics similar to neuronal cells) showed a dose-dependent promotion of neurite outgrowth and nerve regeneration. This effect was mediated by the phosphorylation of v-akt murine thymoma viral oncogene homolog 1 (AKT1).^14^ AKT1 is a serine/threonine kinase that plays a crucial role in the PI3K/protein kinase B/mTOR signaling pathway, which regulates cellular functions such as metabolism, angiogenesis, cell proliferation, and survival. In a rat model of sciatic nerve injury, postoperative low-output FIR accelerates nerve regeneration following end-to-end neurorrhaphy. This therapy results in a larger area of regenerated nerve tissue, increased nerve fiber diameter, and thicker myelin sheaths. These changes contribute to improved functional recovery, enhanced inflammatory responses, and better muscle reinnervation.^15^

### Stress-relieving effects of FIR

In experiments involving acute restraint stress in mice, exposure to FIR mitigated acute restraint stress-induced increases in c-Fos immunoreactivity, oxidative stress, and corticosterone levels by inhibiting JAK2/STAT3 signaling through the induction of glutathione peroxidase.^16^ In a study using a mouse model of depression induced by chronic restraint stress, FIR reduced the levels of cytokines, including interleukin (IL)-1β, IL-6, and tumor necrosis factor-α (TNF-α), in both peripheral tissues and the brain. This reduction helped to mitigate the damage to hippocampal neurons and microglia, thereby alleviating chronic restraint stress-induced depressive-like behaviors in the mice.^17^

### Effects of FIR on animal models of neurological diseases

FIR can effectively prevent impairments in motor coordination, balance, gait, and the connectivity of cerebellar Purkinje cells in transgenic mice with spinocerebellar ataxia type 3 (SCA3). FIR supports cerebellar Purkinje cell survival and promotes autophagy, as reflected by the induction of the autophagy markers microtubule-associated protein light chain 3-II (MAP1LC3, also known as LC3-II) and beclin-1 (also known as Atg6). Concomitantly, FIR reduces p62 and ataxin-3 (ATXN3) accumulation in cerebellar Purkinje cells, suggesting that this may partially contribute to the mechanism of cellular rescue.^18^ The adaptor protein p62 directs waste products to autophagy within the cell, and SCA3 results from an abnormal expansion of a CAG repeat (polyglutamine; polyQ) in *ATXN3*. In a separate study, we created a cell model of SCA3 by transfecting neuroblastoma cells (SK-N-SH) with the pathogenic ATXN3-78Q protein to test whether FIR mediates beneficial effects. FIR-exposed cells expressing the mutant ATXN3-78Q exhibited improved mitochondrial viability and respiratory function and reduced levels of mitochondrial fragmentation, thereby protecting cells from metabolic stress. Moreover, the expression profiles of LC3-II, beclin-1, and p62 suggested that FIR suppresses the autophagy-inhibiting effects observed in ATXN3-78Q-expressing cells.^19^

PD is a neurodegenerative disorder that is characterized by the pathological accumulation and proliferation of α-synuclein, resulting in the death of dopaminergic neurons in the substantia nigra and striatum. A study using A53T α-synuclein-expressing PD model mice (A53T mice) demonstrated that exposure to an FIR ceramic blanket increases cell volume in the striatum and substantia nigra and improves motor performance.^20^

### Effects of electromagnetic waves of other wavelengths on the nervous system

MS is a chronic autoimmune demyelinating disease that affects the central nervous system. In an animal model of experimental autoimmune encephalomyelitis (EAE), which is an experimental version of MS in a controlled setting, it has been observed that although currently approved immunomodulatory and immunosuppressive therapies for MS treatment can slow disease progression, they are unable to completely prevent it. Photobiomodulation induced by 670-nm light reportedly enhances nitric oxide production specific to antigens in heterogeneous lymphocyte populations derived from C57BL/6 mice with myelin/oligodendrocyte glycoprotein-induced EAE—a process partially mediated by immunomodulation. Additionally, 670-nm light-induced photobiomodulation downregulates inducible nitric oxide synthase gene expression in the spinal cords of mice with EAE during disease progression. NIR is 700–1400 nm, meaning that light around 670 nm is not precisely infrared but is rather visible red light (red light) in the boundary region of NIR (deep red). Additionally, B-cell/CLL lymphoma 2 (Bcl-2) anti-apoptotic gene expression is upregulated, the Bcl-2-associated X protein (BAX) ratio is increased, and apoptosis is reduced in the spinal cord of animals throughout the disease course. Nitric oxide has diverse roles in EAE and MS, with evidence suggesting that axonal loss and disability progression in MS may be influenced by nitroso-oxidative stress. Nitric oxide downregulates nitrosative stress in the central nervous system and exerts a protective effect against apoptosis. Together, these findings suggest that this non-invasive treatment may provide neuroprotective effects that produce lasting benefits in MS patients.^21^

A study using 670-nm light-emitting diode arrays on primary cultured neurons demonstrated a partial restoration of cytochrome c oxidase (CcO) activity and a reduction in neuronal cell death. The mechanism of this photobiomodulation involves the upregulation of the CcO enzyme, leading to increased energy metabolism in neurons that have been functionally inactivated by toxins.^22^ Furthermore, graphene-FIR modifies the intestinal microbiota of mice fed a high-fat diet, promoting beneficial bacteria while reducing the levels of harmful bacteria. Graphene-FIR treatment also enhances intestinal barrier function and decreases the levels of the inflammatory cytokines IL-6, IL-1β, and TNF-α in both the peripheral and central nervous systems.^23^

Table 1 summarizes FIR research subjects and the effects of FIR on the nervous system.

## Discussion

All organic matter emits FIR rays. The human body temperature ranges from 36.5 to 37.0°C, and the wavelength of FIR rays emitted from organic matter at this temperature range is approximately 9 μm (9.6 μm at 36.5°C). The human body consists of approximately 37 trillion cells,^24^ which synchronize their vibrations approximately 2000 times per minute to maintain body temperature. Additionally, 65%–70% of the human body is water. Heat from conduction penetrates up to 2 mm below the skin surface, whereas heat from radiation can reach 40 mm below the skin.^2,5^ Among FIR rays, growth rays (8–14 μm) are believed to resonate most effectively with water because their wavelengths are similar to those of the molecules that make up the human body and water. When FIR rays hit an object, their energy is absorbed by water molecules and other macromolecular substances within the object. Improved metabolism leads to more efficient fat burning and enhanced tissue regeneration.^2,5^ Furthermore, it has become clear that some cell types exhibit “non-thermal effects” that cannot be explained by thermal effects alone.^25–27^ Improved blood circulation^28^ also balances the autonomic nervous system,^29^ reducing stress,^30^ promoting mental relaxation,^17^ improving sleep quality,^30^ and assisting in relieving mental fatigue. Improved blood flow around the internal organs also improves the intestinal environment, potentially increasing immunity and affecting the brain through gut–brain interactions.

Thus, FIR increases body temperature and improves blood flow. Blood contains various substances throughout the body that may function as endocrine receptors for tissues, organs, and the brain. Angiogenesis and vascular protection by FIR are essential for maintaining normal neural circuit function in the brain. The blood–brain barrier protects the brain by transporting oxygen and nutrients from blood vessels, excreting unnecessary substances, and blocking harmful substances from entering. It enhances the permeation of various substances, such as glucose, into and out of the brain. The accumulation of stress-related substances can lead to inflammation. Moreover, increased blood flow stimulates glucose metabolism, consequently enhancing glucose uptake in the brain. This results in elevated energy production and metabolism, facilitated by improved mitochondrial activity. Consequently, this process activates neurons and glial cells within the brain. Given its high oxygen utilization, the brain is particularly susceptible to oxidative stress. Oxidative stress harms neurons and tissue in the brain, leading to decreased brain function and cognitive impairment, and is linked to psychiatric disorders.^31–33^ Oxidative stress also leads to inflammation through various mechanisms. Importantly, it is becoming increasingly clear which pathways are targeted by FIR to reduce oxidative stress and inflammation.

Calcium (Ca^2+^) metabolism, stimulated by FIR, is crucial for neuronal functioning. By binding to Ca^2+^, calmodulin (CaM) acts as a Ca^2+^ buffer and interacts with downstream proteins to regulate their activity, and is essential as a Ca^2+^ sensor in Ca^2+^ signaling. In terms of brain function, CaM regulates downstream effector enzymes, including its targets Ca^2+^/CaM-dependent protein kinase II, calcineurin (PP2B), and adenylate cyclase. PP2B is a serine/threonine phosphatase that depends on Ca^2+^ and CaM; it is abundantly expressed in the central nervous system. In the central nervous system, Ca^2+^ induced by synaptic stimulation activates PP2B, thereby allowing it to bind to various receptors and dephosphorylate a variety of substrates. In the brain, it plays a crucial role in regulating Ca^2+^ signaling, which is vital for multiple processes including synapse formation, synaptic plasticity, and neurite outgrowth. It also contributes to memory and learning, as well as to the regulation of intracellular Ca^2+^, gene expression, and apoptosis.^34–37^

In humans, using the DAZZEON αSleep^®^ FIR Blanket for 14 consecutive days reportedly enhances blood circulation, lowers blood pressure, reduces vascular age and arteriosclerosis, increases levels of serotonin and melatonin, improves sleep quality, decreases inflammation, aids in recovery from fatigue, and enhances muscle strength and reaction time.^38^

In conclusion, the present review summarized research reports analyzing the direct effects of FIR on the nervous system. There are numerous reports of the effects of FIR on living organisms in fields other than the nervous system, and the potential for FIR to influence the nervous system is highly promising based on these findings. For example, FIR affects almost all parts of the body, including glucose metabolism,^39^ energy metabolism,^40–42^ antioxidant stress,^43,44^ anti-inflammatory effects,^45,46^ angiogenesis and protective effects,^47,48^ improved blood flow,^49–51^ wound healing,^52,53^ Ca^2+^ regulation,^54,55^ antitumor effects,^56^ lipid metabolism,^57,58^ enzyme activation,^59^ and signal transduction pathways.^60–62^ These phenomena are likely to also occur in the nervous system. Furthermore, although it is difficult to directly identify the effects of FIR on the nervous system in humans, continued research into cellular and animal models is expected to greatly contribute to our understanding of the effects of FIR. Future research may reveal the mechanisms by which FIR influences the nervous system, thereby aiding in our understanding of nervous system diseases and the development of treatment applications and preventive measures.

## Figures and Tables

**Table 1:** FIR research subjects and the effects of FIR on the nervous system

FIR research subjects	Effects of FIR on the nervous system	Reference number
PKCδ KO mice	Inhibits PKCδ phosphorylation by bryostatin 1 Upregulates M1mAChR Nrf2-dependent GSH induction Attenuates MA-induced recognition memory impairment via ERK1/2 phosphorylation	[8]
MA-induced recognition memory impairment mice	Activates Nrf2-dependent GSH synthesis pathway Inhibits PKCδ gene expression, preventing MA-induced memory impairment via ERK1/2 signaling	[9]
AD Tg mice (TgCRND8)	Reduces Aβ burden and tau protein hyperphosphorylation, suppressing neuroinflammation Improves cognitive dysfunction by restoring oxidation-related protein levels through modulation of the JAK2/STAT3 and Nrf2/HO-1 pathways	[10]
AD Tg mice (5xFAD)	Increases NGF expression levels in the hippocampus Increases mature BDNF expression levels in the cerebral cortex	[11]
AD Tg mice (APPswe/PSEN1dE9)	Promotes Aβ phagocytosis via the PI3K/mTOR pathway, resulting in Aβ removal Activates the mitochondrial oxidative phosphorylation pathway, increasing ATP production Promotes extracellular ATP release from Aβ-stimulated microglia Improves learning and memory impairment	[12]
NGF-treated PC12 cells	Promotes neurite outgrowth and nerve regeneration Activates AKT1 phosphorylation	[14]
Sciatic nerve injury rat	Accelerates nerve regeneration and regenerates nerve tissue over a wide area Function is restored by increasing the diameter of nerve fibers and thickening the myelin sheath Promotes nerve repair through inflammatory responses and muscle reinnervation	[15]
Acute restraint stress (ARS) mice	Inhibits JAK2/STAT3 signaling by GPx induction Increases c-Fos-immunoreactivity and oxidative stress by ARS induction Prevents elevated corticosterone levels	[16]
Depression in mice using CRS	Reduces peripheral and brain levels of related cytokines such as IL-1β, IL-6, and TNF-α Reduces damage to hippocampal neurons and microglia Attenuates CRS-induced depressive-like behaviors	[17]
SCA3 Tg mice	Prevents impairments in functions such as motor coordination, balance, and gait, as well as loss of cerebellar Purkinje cell connectivity Supports cerebellar Purkinje cell survival Induces LC3-II and Atg6 and promotes autophagy Decreases accumulation of p62 and ATXN3 in cerebellar Purkinje cells	[18]
SCA3 cell model (SK-N-SH cells transfected with pathogenic ataxin 3-78Q protein)	Prevents decline in mitochondrial viability, improves respiratory function, and reduces mitochondrial fragmentation Protects cells from metabolic stress Suppresses autophagy inhibition by inducing LC3-II, beclin-1, and p62 expression	[19]
PD mice (A53T α-Syn-expressing )	Increases cell volume in the striatum and substantia nigra, improving motor skills	[20]
MS mice with encephalitis (EAE mice)	Downregulates iNOS gene expression in the spinal cord of mice with EAE Upregulates the Bcl-2 anti-apoptotic gene Elevates the Bcl-2:BAX ratio Decreases apoptosis in the spinal cord during the disease course Downregulates nitrosative stress in the central nervous system and its protective effect against apoptosis	[21]
Primary cultured neurons	Partially restores CcO activity and reduces neuronal cell death Increases neuronal energy metabolism	[22]
High fat diet (HFD) mice	Increases beneficial bacteria and decreases harmful bacteria in the intestinal flora Improves intestinal barrier function and decreases the inflammatory cytokines IL-6, IL-1β, and TNF-α in both the peripheral and central nervous systems	[23]

PKCδKO mice: AD model mice with PKCδ knockout; TgCRND8 mice: *APP* transgenic mice harboring a double mutation (K670N/M671L and V717F) associated with familial AD; 5xFAD transgenic mice: transgenic mice overexpressing both mutant human amyloid beta (A4) precursor protein 695 with the Swedish (K670N, M671L), Florida (I716V), and London (V717I) FAD mutations and human presenilin 1 harboring two familial AD mutations, M146L and L286V, which are both associated with early-onset AD; *APP/PS1* knock-in mice: double transgenic mice expressing a chimeric mouse/human amyloid precursor protein (Mo/HuAPP695swe) and a mutant human presenilin 1 (PS1-dE9), both directed to central nervous system neurons; SCA3 transgenic mice: disease model of spinocerebellar ataxia type 3; PD mice (A53T α-Syn-expressing): a mouse model of the brain pathology of PD, created via the unilateral injection of AAV2 hA53T-α-syn viral particles into wild-type mice; MS mice with encephalitis (EAE mice): an animal model of EAE, which is an experimental version of MS in a controlled setting. α-Syn: α-synuclein; Aβ: amyloid β; AD: Alzheimer’s disease; AKT1: v-akt murine thymoma viral oncogene homolog 1; ARS: acute restraint stress; Atg6: beclin-1; ATXN3: ataxin-3; BAX2: Bcl-2-associated X protein; Bcl-2: B-cell/CLL lymphoma 2; BDNF: brain-derived neurotrophic factor; CcO: cytochrome c oxidase; CRS: chronic restraint stress; EAE: experimental autoimmune encephalitis; ERK: extracellular signal-regulated kinase; GPx: glutathione peroxidase; GSH: glutathione; HO-1: heme oxygenase-1; IL: interleukin; iNOS: inducible nitric oxide synthase; JAK2: Janus kinase 2; LC3-II: microtubule-associated protein light chain 3-II; MA, methamphetamine; M1mAChR: M1 muscarinic acetylcholine receptor; MS: multiple sclerosis; mTOR: mechanistic target of rapamycin; NGF: nerve growth factor; Nrf2: NF-E2-related factor 2; PD: Parkinson’s disease; PI3K: phosphoinositide 3-kinase; PKCδ: protein kinase C delta; STAT3: signal transducer and activator of transcription 3; TNF-α: tumor necrosis factor-α.

## Submission declaration

All authors have approved this manuscript, and it has not been published or presented elsewhere in part or in entirety and is not under consideration by another journal.

## References

[1] Cristiano L. Use of infrared-based devices in aesthetic medicine and for beauty and wellness treatments. Infrared Phys Technol 2019; 102: 102991. doi:10.1016/j.infrared.2019.102991

[2] Vatansever F, Hamblin MR. Far infrared radiation (FIR): its biological effects and medical applications. Photonics Lasers Med 2012; 4: 255–266. doi:10.1515/plm-2012-0034 10.1515/plm-2012-0034 PMC3699878 23833705

[3] Li K, Xia L, Liu NF, Nicoli F, Constantinides J, D’Ambrosia C, Lazzeri D, Tremp M, Zhang JF, Zhang YX. Far infrared ray (FIR) therapy: An effective and oncological safe treatment modality for breast cancer related lymphedema. J Photochem Photobiol B 2017; 172: 95–101. doi:10.1016/j.jphotobiol.2017.05.011 10.1016/j.jphotobiol.2017.05.011 28535427

[4] Eells JT, Wong-Riley MT, VerHoeve J, Henry M, Buchman EV, Kane MP, Gould LJ, Das R, Jett M, Hodgson BD, Margolis D, Whelan HT. Mitochondrial signal transduction in accelerated wound and retinal healing by near-infrared light therapy. Mitochondrion 2004; 4: 559–567. doi:10.1016/j.mito.2004.07.033 10.1016/j.mito.2004.07.033 16120414

[5] Karu TI, Pyatibrat LV, Kolyakov SF, Afanasyeva NI. Absorption measurements of cell monolayers relevant to mechanisms of laser phototherapy: reduction or oxidation of cytochrome c oxidase under laser radiation at 632.8 nm. Photomed Laser Surg 2008; 26: 593–599. doi:10.1089/pho.2008.2246 10.1089/pho.2008.2246 19099388

[6] Shui S, Wang X, Chiang JY, Zheng L. Far-infrared therapy for cardiovascular, autoimmune, and other chronic health problems: A systematic review. Exp Biol Med (Maywood) 2015; 240: 1257–1265. doi:10.1177/1535370215573391 10.1177/1535370215573391 PMC4935255 25716016

[7] Yu SY, Chiu JH, Yang SD, Hsu YC, Lui WY, Wu CW. Biological effect of far-infrared therapy on increasing skin microcirculation in rats. Photodermatol Photoimmunol Photomed 2006; 22: 78–86. doi:10.1111/j.1600-0781.2006.00208.x 10.1111/j.1600-0781.2006.00208.x 16606412

[8] Mai HN, Sharma N, Shin EJ, Nguyen BT, Nguyen PT, Jeong JH, Jang CG, Cho EH, Nah SY, Kim NH, Nabeshima T, Kim HC. Exposure to far-infrared rays attenuates methamphetamine-induced recognition memory impairment via modulation of the muscarinic M1 receptor, Nrf2, and PKC. Neurochem Int 2018; 116: 63–76. doi:10.1016/j.neuint.2018.03.009 10.1016/j.neuint.2018.03.009 29572053

[9] Mai HN, Sharma N, Shin EJ, Nguyen BT, Nguyen PT, Jeong JH, Cho EH, Lee YJ, Kim NH, Jang CG, Nabeshima T, Kim HC. Exposure to far-infrared ray attenuates methamphetamine-induced impairment in recognition memory through inhibition of protein kinase C δ in male mice: Comparison with the antipsychotic clozapine. J Neurosci Res 2018; 96: 1294–1310. doi:10.1002/jnr.24228 10.1002/jnr.24228 29476655

[10] Yang W, Yu Q, Wang N, Lam KK, Lin ZX, Xian YF. Far-infrared radiation ameliorates the cognitive dysfunction in an Alzheimer’s disease transgenic mouse via modulating Jak-2/Stat3 and Nrf-2/HO-1 pathways. Neuromolecular Med 2025; 27: 34. doi:10.1007/s12017-025-08860-2 10.1007/s12017-025-08860-2 PMC12081534 40374872

[11] Fukui K, Kimura S, Kato Y, Kohno M. Effects of far infrared light on Alzheimer’s disease-transgenic mice. PLoS One 2021; 16: e0253320. doi:10.1371/journal.pone.0253320 10.1371/journal.pone.0253320 PMC8211253 34138944

[12] Li Q, Peng J, Luo Y, Zhou J, Li T, Cao L, Peng S, Zuo Z, Wang Z. Far infrared light irradiation enhances Aβ clearance via increased exocytotic microglial ATP and ameliorates cognitive deficit in Alzheimer’s disease-like mice. J Neuroinflammation 2022; 19: 145. doi:10.1186/s12974-022-02521-y 10.1186/s12974-022-02521-y PMC9195249 35701825

[13] Wang M, Cao J, Amakye WK, Gong C, Li Q, Ren J. Mid infrared light treatment attenuates cognitive decline and alters the gut microbiota community in APP/PS1 mouse model. Biochem Biophys Res Commun 2020; 523: 60–65. doi:10.1016/j.bbrc.2019.12.015 10.1016/j.bbrc.2019.12.015 31831179

[14] Wang JL, Lin YC, Young TH, Chen MH. Far-infrared ray radiation promotes neurite outgrowth of neuron-like PC12 cells through AKT1 signaling. J Formos Med Assoc 2019; 118: 600–610. doi:10.1016/j.jfma.2018.08.015 10.1016/j.jfma.2018.08.015 30173931

[15] Chen TY, Yang YC, Sha YN, Chou JR, Liu BS. Far-infrared therapy promotes nerve repair following end-to-end neurorrhaphy in rat models of sciatic nerve injury. Evid Based Complement Alternat Med 2015; 2015: 207245. doi:10.1155/2015/207245 10.1155/2015/207245 PMC4333284 25722734

[16] Tran TH, Mai HN, Shin EJ, Nam Y, Nguyen BT, Lee YJ, Jeong JH, Tran HY, Cho EH, Nah SY, Lei XG, Nabeshima T, Kim NH, Kim HC. Repeated exposure to far infrared ray attenuates acute restraint stress in mice via inhibition of JAK2/STAT3 signaling pathway by induction of glutathione peroxidase-1. Neurochem Int 2016; 94: 9–22. doi:10.1016/j.neuint.2016.02.001 10.1016/j.neuint.2016.02.001 26850477

[17] Ling Y, Gao Y, Liu W, Li J, Nie L, Zhu C, Xia Q. The effects and mechanisms of far-infrared ray on depression-like behavior induced by CRS in mice. Brain Res Bull 2025; 225: 111348. doi:10.1016/j.brainresbull.2025.111348 10.1016/j.brainresbull.2025.111348 40252704

[18] Liu SW, Chang JC, Chuang SF, Liu KH, Cheng WL, Chang HJ, Chang HS, Lin TT, Hsieh CL, Lin WY, Hsieh M, Kuo SJ, Liu CS. Far-infrared radiation improves motor dysfunction and neuropathology in spinocerebellar ataxia type 3 mice. Cerebellum 2019; 18: 22–32. doi:10.1007/s12311-018-0936-3 10.1007/s12311-018-0936-3 29725949

[19] Chang JC, Wu SL, Hoel F, Cheng YS, Liu KH, Hsieh M, Hoel A, Tronstad KJ, Yan KC, Hsieh CL, Lin WY, Kuo SJ, Su SL, Liu CS. Far-infrared radiation protects viability in a cell model of Spinocerebellar Ataxia by preventing polyQ protein accumulation and improving mitochondrial function. Sci Rep 2016; 6: 30436. doi:10.1038/srep30436 10.1038/srep30436 PMC4965738 27469193

[20] Carrick FR, Hernandez LSAV, Sugaya K. Amelioration of motor performance and nigrostriatal dopamine cell volume using a novel far-infrared ceramic blanket in an A53T alpha-synuclein transgenic Parkinson’s disease mouse model. Curr Issues Mol Biol 2023; 45: 9823–9837. doi:10.3390/cimb45120613 10.3390/cimb45120613 PMC10742635 38132459

[21] Muili KA, Gopalakrishnan S, Eells JT, Lyons JA. Photobiomodulation induced by 670 nm light ameliorates MOG35-55 induced EAE in female C57BL/6 mice: a role for remediation of nitrosative stress. PLoS One 2013; 8: e67358. doi:10.1371/journal.pone.0067358 10.1371/journal.pone.0067358 PMC3696113 23840675

[22] Wong-Riley MT, Liang HL, Eells JT, Chance B, Henry MM, Buchmann E, Kane M, Whelan HT. Photobiomodulation directly benefits primary neurons functionally inactivated by toxins: role of cytochrome c oxidase. J Biol Chem 2005; 280: 4761–4771. doi:10.1074/jbc.M409650200 10.1074/jbc.M409650200 15557336

[23] Zhang JS, Li S, Cheng X, Tan XC, Huang YL, Dong HJ, Xue R, Zhang Y, Li JC, Feng XX, Deng Y, Zhang YZ. Far-infrared therapy based on graphene ameliorates high-fat diet-induced anxiety-like behavior in obese mice via alleviating intestinal barrier damage and neuroinflammation. Neurochem Res 2024; 49: 1735–1750. doi:10.1007/s11064-024-04133-9 10.1007/s11064-024-04133-9 38530508

[24] Bianconi E, Piovesan A, Facchin F, Beraudi A, Casadei R, Frabetti F, Vitale L, Pelleri MC, Tassani S, Piva F, Perez-Amodio S, Strippoli P, Canaider S. An estimation of the number of cells in the human body. Ann Hum Biol 2013; 40: 463–471. doi:10.3109/03014460.2013.807878 10.3109/03014460.2013.807878 23829164

[25] Yamazaki S, Harata M, Idehara T, Konagaya K, Yokoyama G, Hoshina H, Ogawa Y. Actin polymerization is activated by terahertz irradiation. Sci Rep 2018; 8: 9990. doi:10.1038/s41598-018-28245-9 10.1038/s41598-018-28245-9 PMC6030223 29968762

[26] Yamazaki S, Ueno Y, Hosoki R, Saito T, Idehara T, Yamaguchi Y, Otani C, Ogawa Y, Harata M, Hoshina H. THz irradiation inhibits cell division by affecting actin dynamics. PLoS One 2021; 16: e0248381. doi:10.1371/journal.pone.0248381 10.1371/journal.pone.0248381 PMC8328307 34339441

[27] Sugiyama JI, Tokunaga Y, Hishida M, Tanaka M, Takeuchi K, Satoh D, Imashimizu M. Nonthermal acceleration of protein hydration by sub-terahertz irradiation. Nat Commun 2023; 14: 2825. doi:10.1038/s41467-023-38462-0 10.1038/s41467-023-38462-0 PMC10203368 37217486

[28] Ise N, Katsuura T, Kikuchi Y, Miwa E. Effect of far-infrared radiation on forearm skin blood flow. Ann Physiol Anthropol 1987; 6: 31–32. doi:10.2114/ahs1983.6.31 10.2114/ahs1983.6.31 3675759

[29] Kihara T, Biro S, Imamura M, Yoshifuku S, Takasaki K, Ikeda Y, Otuji Y, Minagoe S, Toyama Y, Tei C. Repeated sauna treatment improves vascular endothelial and cardiac function in patients with chronic heart failure. J Am Coll Cardiol 2002; 39: 754–759. doi:10.1016/S0735-1097(01)01824-1 10.1016/s0735-1097(01)01824-1 11869837

[30] Sato K, Nakamura T, Okimoto H. The thermal effect to organism of ordinary temperature under far-infrared ray radiation sheet. Biomedical Thermology 1998; 18: 160–165 (in Japanese).

[31] Ikeda Y, Saigo N, Nagasaki Y. Direct evidence for the involvement of intestinal reactive oxygen species in the progress of depression via the gut-brain axis. Biomaterials 2023; 295: 122053. doi:10.1016/j.biomaterials.2023.122053 10.1016/j.biomaterials.2023.122053 36821954

[32] Toyoshima M, Jiang X, Ogawa T, Ohnishi T, Yoshihara S, Balan S, Yoshikawa T, Hirokawa N. Enhanced carbonyl stress induces irreversible multimerization of CRMP2 in schizophrenia pathogenesis. Life Sci Alliance 2019; 2: e201900478. doi:10.26508/lsa.201900478 10.26508/lsa.201900478 PMC6781483 31591136

[33] Nunomura A, Castellani RJ, Zhu X, Moreira PI, Perry G, Smith MA. Involvement of oxidative stress in Alzheimer disease. J Neuropathol Exp Neurol 2006; 65: 631–641. doi:10.1097/01.jnen.0000228136.58062.bf 10.1097/01.jnen.0000228136.58062.bf 16825950

[34] Hudmon A, Schulman H. Neuronal CA2+/calmodulin-dependent protein kinase II: the role of structure and autoregulation in cellular function. Annu Rev Biochem 2002; 71: 473–510. doi:10.1146/annurev.biochem.71.110601.135410 10.1146/annurev.biochem.71.110601.135410 12045104

[35] Meyer T, Hanson PI, Stryer L, Schulman H. Calmodulin trapping by calcium-calmodulin-dependent protein kinase. Science 1992; 256: 1199–1202. doi:10.1126/science.256.5060.1199 10.1126/science.256.5060.1199 1317063

[36] Stemmer PM, Klee CB. Dual calcium ion regulation of calcineurin by calmodulin and calcineurin B. Biochemistry 1994; 33: 6859–6866. doi:10.1021/bi00188a015 10.1021/bi00188a015 8204620

[37] Winder DG, Sweatt JD. Roles of serine/threonine phosphatases in hippocampal synaptic plasticity. Nat Rev Neurosci 2001; 2: 461–474. doi:10.1038/35081514 10.1038/35081514 11433371

[38] Lee MC, Ho CS, Hsu YJ, Kan NW, Fei CY, Yang HJ, Huang CC. The impact of DAZZEON αSleep^®^ far-infrared blanket on sleep, blood pressure, vascular health, muscle function, inflammation, and fatigue. Clocks Sleep 2024; 6: 499–516. doi:10.3390/clockssleep6030033 10.3390/clockssleep6030033 PMC11417803 39311228

[39] Li S, Miao XY, Zhang JS, Wei DD, Dong HJ, Xue R, Li JC, Zhang Y, Feng XX, Li J, Zhang YZ. Far-infrared therapy promotes exercise capacity and glucose metabolism in mice by modulating microbiota homeostasis and activating AMPK. Sci Rep 2024; 14: 16314. doi:10.1038/s41598-024-67220-5 10.1038/s41598-024-67220-5 PMC11251280 39009692

[40] Seo Y, Kim YW, Lee D, et al. Far-infrared rays enhance mitochondrial biogenesis and GLUT3 expression under low glucose conditions in rat skeletal muscle cells. Korean J Physiol Pharmacol 2021; 25: 167–175. doi:10.4196/kjpp.2021.25.2.167 10.4196/kjpp.2021.25.2.167 PMC7893496 33602887

[41] Hsu YH, Chen YC, Chen YW, Chiu TH, Kuo YT, Chen CH. Far-infrared radiation prevents decline in β-cell mass and function in diabetic mice via the mitochondria-mediated Sirtuin1 pathway. Metabolism 2020; 104: 154143. doi:10.1016/j.metabol.2020.154143 10.1016/j.metabol.2020.154143 31927009

[42] Chen CH, Chen TH, Wu MY, Chou TC, Chen JR, Wei MJ, Lee SL, Hong LY, Zheng CM, Chiu IJ, Lin YF, Hsu CM, Hsu YH. Far-infrared protects vascular endothelial cells from advanced glycation end products-induced injury via PLZF-mediated autophagy in diabetic mice. Sci Rep 2017; 7: 40442. doi:10.1038/srep40442 10.1038/srep40442 PMC5223182 28071754

[43] Kawaura A, Tanida N, Kamitani M, Akiyama J, Mizutani M, Tsugawa N, Okano T, Takeda E. The effect of leg hyperthermia using far infrared rays in bedridden subjects with type 2 diabetes mellitus. Acta Med Okayama 2010; 64: 143–147. doi:10.18926/amo/32849 10.18926/AMO/32849 20424670

[44] Wang HW, Huang TS, Lo HH, Huang PH, Lin CC, Chang SJ, Liao KH, Tsai CH, Chan CH, Tsai CF, Cheng YC, Chiu YL, Tsai TN, Cheng CC, Cheng SM. Deficiency of the microRNA-31-microRNA-720 pathway in the plasma and endothelial progenitor cells from patients with coronary artery disease. Arterioscler Thromb Vasc Biol 2014; 34: 857–869. doi:10.1161/atvbaha.113.303001 10.1161/ATVBAHA.113.303001 24558106

[45] Hubbard AK, Rothlein R. Intercellular adhesion molecule-1 (ICAM-1) expression and cell signaling cascades. Free Radic Biol Med 2000; 28: 1379–1386. doi:10.1016/s0891-5849(00)00223-9 10.1016/s0891-5849(00)00223-9 10924857

[46] Lin CC, Liu XM, Peyton K, Wang H, Yang WC, Lin SJ, Durante W. Far infrared therapy inhibits vascular endothelial inflammation via the induction of heme oxygenase-1. Arterioscler Thromb Vasc Biol 2008; 28: 739–745. doi:10.1161/atvbaha.107.160085 10.1161/ATVBAHA.107.160085 PMC2748062 18202320

[47] Jeong YM, Kim W. FIR-preconditioning promotes Akt-mTOR-exosome manufacture in cooperation with MITF to boost resilience of rat bone marrow-derived stem cells. Heliyon 2023; 9: e15003. doi:10.1016/j.heliyon.2023.e15003 10.1016/j.heliyon.2023.e15003 PMC10130773 37123908

[48] Rau CS, Yang JC, Jeng SF, Chen YC, Lin CJ, Wu CJ, Lu TH, Hsieh CH. Far-infrared radiation promotes angiogenesis in human microvascular endothelial cells via extracellular signal-regulated kinase activation. Photochem Photobiol 2011; 87: 441–446. doi:10.1111/j.1751-1097.2010.00853.x 10.1111/j.1751-1097.2010.00853.x 21143604

[49] Tu YP, Chen SC, Liu YH, Chen CF, Hour TC. Postconditioning with far-infrared irradiation increases heme oxygenase-1 expression and protects against ischemia/reperfusion injury in rat testis. Life Sci 2013; 92: 35–41. doi:10.1016/j.lfs.2012.10.019 10.1016/j.lfs.2012.10.019 23142244

[50] Chen CH, Chen MC, Hsu YH, Chou TC. Far-infrared radiation alleviates cisplatin-induced vascular damage and impaired circulation via activation of HIF-1α. Cancer Sci 2022; 113: 2194–2206. doi:10.1111/cas.15371 10.1111/cas.15371 PMC9207382 35411640

[51] Shimizu N, Asatsuma-Okumura T, Yamamoto J, Yamaguchi Y, Handa H, Ito T. PLZF and its fusion proteins are pomalidomide-dependent CRBN neosubstrates. Commun Biol 2021; 4: 1277. doi:10.1038/s42003-021-02801-y 10.1038/s42003-021-02801-y PMC8586336 34764413

[52] Chiu HW, Chen CH, Chen YJ, Hsu YH. Far-infrared suppresses skin photoaging in ultraviolet B-exposed fibroblasts and hairless mice. PLoS One 2017; 12: e0174042. doi:10.1371/journal.pone.0174042 10.1371/journal.pone.0174042 PMC5354422 28301572

[53] Kimura I, Yamamoto T, Nakamura K, Uenishi T, Asai T, Kita M, Kanamura N. Effects of far infrared radiation by isotropic high-density carbon on the human oral mucosa. Arch Oral Biol 2018; 94: 62–68. doi:10.1016/j.archoralbio.2018.06.011 10.1016/j.archoralbio.2018.06.011 29979974

[54] Kim HY, Yu Y, Oh SY, Wang KK, Kim YR, Jung SC, Kim HS, Jo I. Far-infrared irradiation inhibits adipogenic differentiation and stimulates osteogenic differentiation of human tonsil-derived mesenchymal stem cells: role of protein phosphatase 2B. Cell Physiol Biochem 2019; 52: 240–253. doi:10.33594/000000018 10.33594/000000018 30816672

[55] Park JH, Lee S, Cho DH, Park YM, Kang DH, Jo I. Far-infrared radiation acutely increases nitric oxide production by increasing Ca(2+) mobilization and Ca(2+)/calmodulin-dependent protein kinase II-mediated phosphorylation of endothelial nitric oxide synthase at serine 1179. Biochem Biophys Res Commun 2013; 436: 601–606. doi:10.1016/j.bbrc.2013.06.003 10.1016/j.bbrc.2013.06.003 23756809

[56] Cho DH, Lee HJ, Lee JY, Park JH, Jo I. Far-infrared irradiation inhibits breast cancer cell proliferation independently of DNA damage through increased nuclear Ca(2+)/calmodulin binding modulated-activation of checkpoint kinase 2. J Photochem Photobiol B 2021; 219: 112188. doi:10.1016/j.jphotobiol.2021.112188 10.1016/j.jphotobiol.2021.112188 33901880

[57] Xu T, Fu H, Zhao W, Shan S. Far-infrared radiation alleviates steatohepatitis and fibrosis in metabolic dysfunction-associated fatty liver disease. Sci Rep 2024; 14: 19292. doi:10.1038/s41598-024-69053-8 10.1038/s41598-024-69053-8 PMC11336198 39164313

[58] Park JH, Oh SY, Jung SC, Song TJ, Jo I. Far-infrared irradiation increases the uptake of LDL cholesterol by downregulating PCSK9 through the activation of TRPV4 calcium channels in HepG2 cells. Biochem Biophys Res Commun 2024; 723: 150187. doi:10.1016/j.bbrc.2024.150187 10.1016/j.bbrc.2024.150187 38850809

[59] Chiang IN, Pu YS, Huang CY, Young TH. Far infrared radiation promotes rabbit renal proximal tubule cell proliferation and functional characteristics, and protects against cisplatin-induced nephrotoxicity. PLoS One 2017; 12: e0180872. doi:10.1371/journal.pone.0180872 10.1371/journal.pone.0180872 PMC5513434 28715443

[60] Heldin CH, Miyazono K, ten Dijke P. TGF-beta signalling from cell membrane to nucleus through SMAD proteins. Nature 1997; 390: 465–471. doi:10.1038/37284 10.1038/37284 9393997

[61] Xie Y, Shi X, Sheng K, Han G, Li W, Zhao Q, Jiang B, Feng J, Li J, Gu Y. PI3K/Akt signaling transduction pathway, erythropoiesis and glycolysis in hypoxia (Review). Mol Med Rep 2019; 19: 783–791. doi:10.3892/mmr.2018.9713 10.3892/mmr.2018.9713 PMC6323245 30535469

[62] Keppler-Noreuil KM, Parker VE, Darling TN, Martinez-Agosto JA. Somatic overgrowth disorders of the PI3K/AKT/mTOR pathway & therapeutic strategies. Am J Med Genet C Semin Med Genet 2016; 172: 402–421. doi:10.1002/ajmg.c.31531 10.1002/ajmg.c.31531 PMC5592089 27860216

